# Gut Microbiota: A Promising Milestone in Enhancing the Efficacy of PD1/PD-L1 Blockade Therapy

**DOI:** 10.3389/fonc.2022.847350

**Published:** 2022-02-16

**Authors:** Yuqing Zhou, Zhaoxia Liu, Tingtao Chen

**Affiliations:** ^1^Department of Obstetrics and Gynecology, The Second Affiliated Hospital of Nanchang University, Nanchang, China; ^2^Queen Mary School, Nanchang University, Nanchang, China; ^3^National Engineering Research Center for Bioengineering Drugs and Technologies, Institute of Translational Medicine, Nanchang University, Nanchang, China

**Keywords:** cancer, immunotherapy, PD1/PD-L1, gut microbiome, probiotics

## Abstract

In the past few decades, immunotherapy has emerged as one of the most promising strategies among current treatments of cancer. In particular, the field of PD1/PD-L1 inhibitors has been boosted, widely applied into clinical practice with potent therapeutic efficacy and remarkable survival benefits on various cancers such as melanoma, non-small cell lung cancer (NSCLC), and urothelial carcinoma (UC). However, the application of PD1/PD-L1 blockade therapy is still quite restricted because of unexpected toxicities, limited response rate, as well as associated resistance. In consequence, searching for potential strategies that possibly resolve the existing limitations and enhance the therapeutic responsiveness of PD1/PD-L1 blockade is of great significance. Fortunately, the gut microbiome has been demonstrated to serve as a pivotal regulator in anti-PD1/PD-L1 therapy, providing an applicable tool to improve anti-PD1/PD-L1 clinical efficacy. In this review, we summarized published advancements about how microbiota modulated in anti-PD1/PD-L1 therapy and illustrated its underlying mechanisms, giving insights into putative manipulation of gut microbiota to facilitate PD1/PD-L1 blockade.

## Introduction

Cancer is a multistep disorder that arises through a combination of genetic and epigenetic alterations, which ultimately facilitate malignant transformation and cell immortality (1). According to the estimation of Disability-Adjusted Life Years (DALYs), malignancies possess the most considerable global burden amongst all human diseases, which are estimated to become the leading cause of mortality by the end of 2060 (1, 2). To date, surgery, chemotherapy, and radiotherapy remain the mainstream for standard cancer strategies; however, risks for post-therapeutic side effects, including accidental infection, immunity suppression, and multi-drug resistance (MDR) still exist when applying these current methods (3–5). Therefore, concerning the undesirable reactions of conventional cures, the focus has been shifted to more precisely targeted cancer immunotherapies, with the hope to elude the above by-effects, emerging as one of the standard anti-tumor therapies in clinical fields (6, 7).

In terms of immunotherapies, immune checkpoint inhibitors (ICIs) are regarded as one of the most crucial counterparts in treating a number of advanced cancers, especially for the application of programmed cell death 1 (PD1) and programmed cell death 1 ligand 1 (PD-L1) inhibitors (3, 6, 8). The monoclonal antibodies against PD1 and PD-L1 specifically target and block these two immunoregulatory sites, largely unleashing the immunotolerance and strengthening the antitumor immunity *via* impeding the inhibitory signaling pathways, derepressing the co-stimulatory signals, and accelerating T cell re-activation. So far, PD1/PD-L1 monoclonal antibodies (mAbs) have achieved encouraging results in a series of clinical trials (3, 4, 6, 8–11). In particular, atezolizumab (anti-PD-L1 mAb), nivolumab(anti-PD1 mAb), and pembrolizumab (anti-PD1 mAb) have already been approved with durable clinical response and prolonged overall survival (OS), reaching clinics for the treatment of melanoma, non-small cell lung cancer (NSCLC), and renal cell carcinoma (RCC) (6, 12). Despite these advancements, the usage of ICIs still faces great challenges, including unexpected adverse effects, slow-onset time as well as the limited responsive ratio (4, 7, 13). Therefore, there is an urgent need to uncover reasons for underlying unresponsiveness and accurately target the putative benefiters of PD1/PD-L1 blockade among overall patients. Fortunately, a growing number of studies have evidenced that the gut microbiome could contribute significantly to enhancing anti-PD1/PD-L1 therapeutic responses (14–16).

The gut microbiome refers to the diverse species of nearly 10^14^ microorganisms that inhabit along the intestinal lumen, including bacteria, fungi, viruses, protozoa, and archaea (17, 18). Over recent years, accumulating evidence has highlighted that the gut microbes have actively involved in the initiation, progression, as well as treatment of a variety of diseases such as diabetes, inflammatory bowel disease (IBD), and even cancers (19). Furthermore, it was also emphasized that the gut flora probably function in the modulation of PD1/PD-L1 blockade through bacteria translocation or sending bacteria-derived molecules to enhance antigenicity and endeavor anti-tumor immune response, which was proved by evidence that certain microbiota types were explicitly found to be enriched in ICI effective patients while some corresponded with the non-responsiveness of PD1/PD-L1 blockade (20–24). In this case, we would discuss the role of the gut microbiome in anti-PD1/PD-L1 therapy and its putative mechanisms, manipulation strategies as well as the prospect of clinical application in this review.

## Harness the Power of Host Immune System to Combat Cancer: PD1/PD-L1 Blockade in Immunotherapy

As a multifactorial disease, cancer is the result of genetic susceptibility and oncogenic stimulants (25). In spite of progressive understanding of cancer etiology and bioactivities, surgery, chemotherapy, and radiotherapy still comprise the standard treatments in a majority of invading tumors (26). Nevertheless, unprecise target selectivity and treatment-induced toxicities remain the two most critical issues of these traditional therapeutic methods (25). Notably, in the past few decades, the idea to harness the power of the immune system and revive the compromised anti-cancer immunosurveillance has come into sight, pushing forward the development of cancer immunotherapy. A variety of approaches were included in the field of immunotherapies, ranging from active stimulation (reactivate the immune effectors) to passive ones (counteract the inhibitory cellular mechanisms), with PD1/PD-L1 inhibitors becoming one of the most applied therapy in treating various solid and hematologic tumors (3, 4).

PD1/PD-L1 inhibitors are monoclonal antibodies that specifically block membrane receptors that are involved in the immunosuppressive signaling pathway, thus unleashing the immune tolerance of tumor-infiltrating lymphocytes (TILs) within the tumor microenvironment (TME) and invigorating endogenous antitumor response to tumor lesions (27). PD1 is a co-inhibitory receptor predominantly expressed on several immune cells, including activated T cells, B cells, natural killer (NK) cells, and dendritic cells (DCs) (28, 29). During T cell activation, the PD1 expression is provoked by the cytokines (IL-2,7,15,21) after the antigen-specific engagement of T cell receptors (TCR) with major histocompatibility complex (MHC), essentially prohibiting the hyperactivation of self-reactive T cells. In general, there are two corresponding ligands for PD1, named PD-L1 and PD-L2; both are continuously expressed by antigen-presenting cells (APCs) to sustain immune tolerance and refrain from excessive autoimmunity and improper peripheral damage (30, 31). The exact role of PD-L2 in cancer immunity is yet unstated. However, the peripheral binding of PD1/PD-L1 is responsible for the resistance of cytotoxic T-lymphocytes (CTLs) mediated cytolysis as well as Fas-induced cellular apoptosis (32–36). Within the TME, the release of IFN-γ and stimulation of oncogenic drivers could provoke PD-L1 overexpression on tumor cells through inhibiting PI3K-AKT and Ras-Raf-MEK-ERK mediated pathways (37). Therefore, the subsequent binding of PD1 to PD-L1 serves as a brake for T cell activation in the tumor bed, driving T cell exhaustion, apoptosis, and neutralization processes, thus leading to tumor cell survival and unlimited proliferation (38). Notably, PD1/PD-L1 blockers has also achieved significant clinical efficacy against treating various solid and hematologic malignancies. Correspondingly, there are several clinically available PD1/PD-L1 inhibitors approved by FDA for cancer treatment ranging from melanoma to NSCLC, including but not restricted to pembrolizumab, nivolumab, atezolizumab, and avelumab. More importantly, during early phase II trials, the single agent alone yielded encouraging clinical results (39). The atezolizumab alone met its expected endpoint with prominent survival benefits and adequate safety profiles compared to applying chemotherapeutic docetaxel (40). In addition, the anti-PD1 nivolumab, which was designated as the frontline pharmaceutics for melanoma treatment, showed favorable clinical responses with durable sustaining effects and low relapse rate compared to conventional chemotherapies (41). Combinatorial therapy of anti-CTLA-4 and anti-PD-L1 blockade displayed more evident tumor regression in approximately 50% of patients with advanced melanoma, in some cases, more than 80% of total patients were presented with disease remission and long-term free survival (42, 43).

Though PD1/PD-L1 blockade has displayed significant clinical results, it also accompanies a spectrum of toxic events (44). Especially with anti-PD1/PD-L1 therapy, immune-related adverse events (irAEs) are the most frequently occurring types (44–46). IrAEs in PD1/PD-L1 inhibition commonly present with general systemic toxicity such as fatigue, headache, fever, nausea, diarrhea, rash, pruritus, etc. (47). It could also manifest with specific organ destruction, with the skin, thyroid gland, pituitary gland, liver, lung, and GI tract being the most susceptible sites (44, 45). In short, these all underline the necessity of multidisciplinary collaboration and consensus monitoring of irAEs. Interestingly, in recent years, mounting evidence has proposed that microbiota could mitigate the PD1/PD-L1 related toxicities to some extent, further indicating its potential role in modulating the effectiveness and prognosis of anti-PD1/PD-L1 therapy.

## The Interplay Between Gut Microbiome and Host Immunity

The symbionts residing in the gastrointestinal tract contribute significantly to sustaining the homeostasis and overall health of the host. As a pivotal regulator of the digestive system, gut commensals actively participate in the digestion of nutrients, fermentation of dietary fibers, synthesis of necessary vitamins as well as competition for pathogenic invasion, thus maintaining the stability of the gut microenvironment (48). Consequently, disruption of the balance of microbial configuration may result in the break out of numerous diseases, even cancer (27, 48). In particular, specific bacteria species are proved to involve in the oncogenic process to favor tumor immortality. For instance, enterotoxigenic *Bacteroides fragilis* facilitates colorectal cancer progression by generating metalloproteinase, an enzyme that specifically ruins the integrity of the gut mucosal barrier, which in turn increases the exposure risks for pathogens and establishes an immunodeficient gut microenvironment (48). Apart from driving oncogenicity, the intestinal flora is also estimated to influence host immunity multifacetedly. Recently, it has been recognized that gut dysbiosis is intimately associated with the initiation of various immunological disorders such as IBD, celiac disease, rheumatoid arthritis, type-1 diabetes as well as asthma (19). Notably, the gut microbiota is believed to hold complicated crosstalk with the host immune system both locally and systemically, which also hints at its therapeutic effect in manipulating the immunological responses of PD1/PD-L1 blockade (18, 49–53).

The gut microbiota primarily regulates the local host immunity by establishing the competent mucosal immune system, which is referred to as the “second” immune system with unique structures and independent features (54). The physical epithelial barrier is covered with a thick layer of mucus, which is comprised of mucus proteins produced by goblet cells, mucins enriched with antimicrobial peptides as well as immunoglobulin A secreted by B cells (49). The defense of the mucus layer could protect the internal intestinal wall from bacterial adhesion, invasion, and colonization (54). In addition, the gut-associated lymphoid tissues residing in the lamina propria are also crucial counterparts in the mucosal immune system, consisting of groups of histological lymphoid tissues (Peyer’s patches, lymphoid follicles, and mesenteric lymph nodes) and dispersing lymphocytes (DCs, T cells, and B cells, etc.) (54). Generally, the gut microbiota executes its modulatory role in the local gut immunity through the following three manners: (1) regulate the regional B cell function *via* promoting IgA secretion (55), (2) moderate the differentiation of Th17 cells and Treg cells to organize the balance between inflammatory response and immune tolerance (56), (3) modify the function of γδ intraepithelial cell for invasive signal detection (57). In short, the gut microbiome could mobilize the mucosal effector cells and promote the secretion of associated immunomodulatory factors, thus actively participating in the regeneration and maintenance of the mucosal immune system.

Nonetheless, the effect of microbiota is not only limited to completing the localized immunity of the gut but also comes into play at distant sites to influence the overall immune tone (18, 49). Systemically, the gut microbiota has been validated to produce and release microbiota-derived molecular substances into blood circulation to influence the immune responses of distant tissues and organs, managing the development and mobilization of systemic immune cells (DC priming, lymphocyte homing, recirculation, and cross-reactivation) and recognizing signals from Toll-like receptors, thus altering the immune response of extraintestinal diseases (18, 49, 54). In brief, the colonization of gut bacteria is indispensable during the maturation of the host immune system, playing an essential part in maintaining intestinal mucosal homeostasis *via* protecting the integrity of the intestinal barrier and shaping a competent immune system through the systemic mobilization of immune effectors.

## Gut Microbiota Modulates the Efficacy of Anti-PD1/PD-L1 Immunotherapy

Unlike other cancer therapies, PD1/PD-L1 inhibitory immunotherapy mainly functions *via* specifically occluding the immunoinhibitory PD1 or PD-L1 sites, thus boosting the endogenous host immunity. Considering the previous work of the synergistic effects of *Bacteroides fragilis* on CTLA-4 blockers, it is also estimated that the therapeutic outcome of anti-PD1/PD-L1 therapy could also be drastically influenced by microbiota, which has been verified in both preclinical and clinical studies (**Table 1**).

**Table 1:** Regulatory role of gut microbiota in anti-PD1/PD-L1 therapy.

Bacteria	Applied anti-PD1/PD-L1 immunotherapy	Preclinical and clinical cohort	Modulatory effects on anti-PD1/PD-L1 therapeutic responses	Possible Mechanisms of Associated Microbiota	References
*Bifidobacterium* spp.	Anti-PD-L1 blockade	Mice model bearing melanoma	Anti-tumor effects, preventing tumor growth and expansion Synergistic role with anti-PD-L1 therapy	Inducing DCs maturation and activation Increasing accumulation of CD8^+^ T cells in tumor beds	Sivan et al. (20)
*Akkermansia muciniphila*	Anti-PD1 blockade	GF or ATB treated mice Patients with advanced NSCLC, RCC, or UC	Potent clinical response in responders receiving anti-PD1 therapy but not in non-responders Facilitating anti-PD1 therapy	Motivating DCs, promoting IL-12 production Recruitment of CD4^+^ CCR9^+^ T cells and CD4^+^ CXCR3^+^ T cells into TME, reducing Tregs ratio	Routy et al. (22)
*Bifidobacterium longum*, *Enterococcus faecium*, and *Collinsella aerofaciens*	Anti-PD1 blockade	Metastatic melanoma patients	Improving the tumor control in responders Enhanced efficacy of anti-PD1 blockade	Enhanced DCs function and greater Th1 cell responses Decreased Tregs in the periphery	Matson et al. (58)
*Ruminococcaceae/Faecalibacterium/Clostridiales*	Anti-PD1 blockade	Advanced melanoma patients	Responders present with boosted anti-tumor immunity Enhanced anti-PD1 therapeutic responses in responders	Elevating the level of effector CD4^+^ and CD8^+^ T cells in peripheral blood and tumor bed Decreasing the number of Tregs and MDSCs	Gopalakrishnan et al. (21)
*Alistipes putredinis, Bifidobacterium longum*, and *Prevotella copri*	Anti-PD1 blockade	Chinese NSCLC patients	Higher microbiome diversity correspond with prolonged PFS in patients Synergistic function in anti-PD1 therapy	Increasing the aggregation of tumor infiltrating CD8^+^ T cells in the TME Promoting memory T cell and NK cell function	Jin et al. (23)

PD1, programmed cell death 1; PD-L1, programmed cell death 1 ligand 1; GF, germ-free; ATB, antibiotics; NSCLC, non-small-cell lung cancer; RCC, renal cell carcinoma; UC, urothelial carcinoma; PFS, progression-free survival; DCs, dendritic cells; TME, tumor microenvironment; Tregs, regulatory T cells; MDSCs, myeloid-derived suppressor cells; NK cell, natural killer cell.

**Figure 1:**
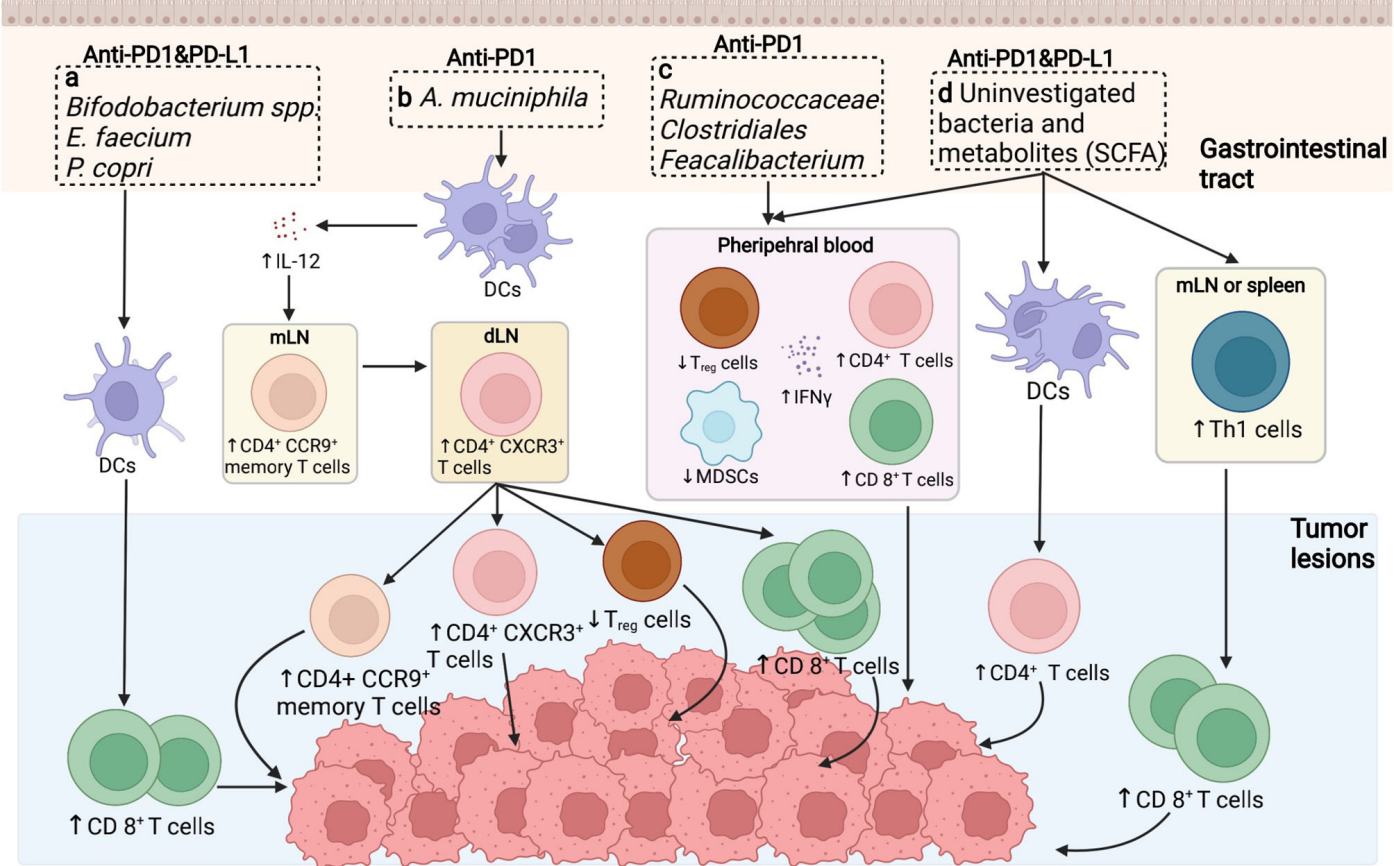
Putative mechanisms concerning the role of the gut microbiome in anti-PD1/PD-L1 immunotherapy. **(A)** In preclinical murine models, the abundance of *Bifidobacterium* spp./*Enterococcus faecium* etc. was shown to increase the cytotoxic T cell function in cancerous sites to facilitate tumor killing. **(B)** The enrichment of *Akkermansiacea muciniphila* in anti-PD-L1 responders is correlated with enhanced dendritic cells (DCs) activation, thus provoking IL-12 secretion, promoting the trafficking of CD4^+^ CCR9^+^ memory T cell and CD4^+^ CXCR3^+^ T cells from mesentery lymph nodes (mLNs) to tumor draining lymph nodes (dLNs), ultimately enhancing anti-tumor effect by motivating effector T cells. **(C)** *Ruminococcaceae/Clostridales/Feacalibacterium* in the GI tract mediate in anti-tumor effect *via* enhancing the ratio of CD4^+^ and CD8^+^ T cell ratio while downregulating the activity of regulatory T cells and myeloid derived suppressor cells (MDSCs). **(D)** Bacteria themselves and associated metabolites are also potential regulators in anti-PD1/PD-L1 therapy, primarily *via* driving Th1 cell differentiation peripherally, potentiating DCs function as well as diminishing circulating regulatory T cells (T_regs_), thus ameliorating immunosuppression and reinforcing immune activation.

The immunomodulatory role of gut microbiota in PD1/PD-L1 inhibiting therapy was firstly investigated in preclinical murine models. Back in 2015, Sivan et al. explored the stimulatory role of *Bifidobacterium* (including *Bifidobacterium breve, Bifidobacterium longum*, and *Bifidobacterium adolescentis*) in assisting the anti-tumor response elicited by anti-PD-L1 mAbs (20). The abundance of *Bifidobacterium* was proved to sufficiently reinforce the cytotoxic T cell response and impede the tumor growth in melanoma-bearing mice primarily *via* enhancing DCs function (20). As Sivan et al. stated, the enrichment of *Bifidobacterium* in responders was validated to upregulate the gene expression in DCs, increase cytokine production, drive DCs maturation and enhance CD8^+^ T cells priming and accumulation in the TME. Meanwhile, the threshold for DC activation declines while IFN-γ production and TILs proliferation increase, allowing for mobilized antigen-presenting ability, upregulated circulating lymphocytes recruitment, and robust effector cell priming, together resulting in immune potentiation and tumor regression (**Figure 1A**) (20, 59).

Furthermore, four other clinical analogous studies of the human microbiome in advanced tumor patients to identify the immunoregulatory role of specific bacteria genres in PD1/PD-L1 blockade were carried out in the following years. In the study of Routy et al., the antibiotic intervention was explored and believed to largely perturb the anti-PD-L1 therapeutic efficacy caused by gut dysbiosis, contributing to the subsequential relapse and shortened survival rate in NSCLC and RCC patients (22). However, the specific enrichment of *Akkermansia muciniphila* extracted in responders could essentially reverse this disadvantageous circumstance, bringing in overt effector T cell action. *A. muciniphila* is estimated to promote tumor-killing activity in an IL-12 dependent manner mediated *via* DCs motivation. Moreover, exposure to *A. muciniphila* is correlated with enhanced trafficking of CC-chemokine receptor 9 (CCR9)-expressing T helper cells into mesenteric lymph nodes, following the accumulation of CD4^+^ CXCR3^+^ T cells into tumor-draining lymph nodes, finally leading to enhanced cytotoxic T cell response as well as reduced regulatory T cell ratio in the TME (**Figure 1B**) (20, 60). In consistent with the above study, concerning the melanoma patient cohort, Matson et al. revealed that the enrichment of *Bifidobacterium longum*, *Enterococcus faecium*, and *Collinsella aerofaciens* was positively correlated with augmented systemic T cell response, optimized tumor control, and improved immune-potentiating effect of anti-PD1 (**Figure 1A**) (58). On the contrary, Gopalakrishnan et al. reported significant divergence in the multiplicity and composition of the gut microbes in responders, with *Clostridiales/Ruminococcaceae/Faecalibacterium* being the most colonized strains. The collection of *Faecalibacterium* spp. enriched in the responders of anti-PD1 treatment was in positive correlation with extended progression-free survival (PFS), in parallel with increased frequency of circulating CD4^+^ and CD8^+^ T cells and stabilized cytokine action in peripheral blood; whereas *Bacteroides* enriched in the non-responders failed to do so, increasing the recurrence risks instead (**Figure 1C**) (21). Conversely, the immunosuppressive circulatory cells such as regulatory T cells (Tregs) and myeloid-derived suppressor cells (MDSCs) were observed to be relatively reduced to some extent (**Figure 1C**) (21, 59). Besides, in terms of the cohort of Chinese NSCLC patients, Jin et al. investigated the interrelationship between the gut microbiota configuration and anti-PD1 blockade efficacy, illustrating another distinct bacterial make-up in well responders, consisting of *Alistipes putredinis, Bifidobacterium longum*, and *Prevotella copri* (**Figure 1A**) (23).

Presumably, specific bacteria translocation and bacteria-derived metabolites are considered as other putative mechanisms to facilitate anti-PD1/PD-L1 therapy (20, 24, 61). Despite that direct bacteria translocation was not evidently marked during PD1/PD-L1 targeting therapy, it might also facilitate anti-PD1/PD-L1 response due to the amelioration of peripheral immune tolerance (62). Gaining access to the mesenteric lymph nodes, spleen, and tumor lesions crossing the impaired gut barrier, translocated bacteria promote the differentiation of Th1 cells in secondary immune organs in the periphery, later unleashing immunosuppression and activating lymphocytes recirculation to facilitate PD1 blockade in tumor beds (**Figure 1D**) (63, 64). Besides, microbial metabolites could also act as immunomodulators, with short-chain fatty acid (SCFA) being one of the most investigative bioactive byproducts (61). Primarily, SCFA could be utilized by intestinal epithelial cells as a source of energy; in turn, these metabolites also exert a broad range of effects on host immunity, mainly mediating in the processes of cytokine production, antigen-presenting activities, and Treg differentiation, eventually affecting the anti-PD1/PD-L1 efficacy (**Figure 1D**) (65). Moreover, Geller et al. reported that viable bacteria were found to colonize in human pancreatic ductal adenocarcinoma, suggesting that tumor microbiota might exert molecular mimicry on tumor cells and influence the PD1/PD-L1 blockade outcomes as well (not confirmed yet) (66). Nevertheless, evidence regarding this theory is inconsistent among studies, which requires validating experiments to further confirm its contribution towards augmented immunostimulatory effects (67).

Excitingly, apart from directly affecting ICI outcome, divergence in the microbial composition may also provide clinical value for assessing the ICI-derived toxicity scores. Evidence from both preclinical and clinical studies has characterized the contribution of gut microbes on regulating the occurrence of ICI-derived adverse effects. Considering that anti-CTLA-4-treated melanoma patients rich in *Bacteroidetes* harness a lower risk for colitis mediated by the Tregs differentiation process, efforts to explore whether the anti-PD1/PD-L1 related toxicity could also be ameliorated in a bacteria-dependent manner are currently on the way (68). Based on the available literature, bacteria belonging to the *Ruminococaceae* family were identified to optimize both response and irAEs towards ICI therapy (69). In addition, although *Bacteriodales* represented a typical sign of non-responders, increased colonization of this genre was found to correlated with a lower frequency of ICI-induced autoimmune disease (69, 70).

## Challenges, Clinical Application, and Possible Strategies of Gut Microbiota Applied in PD1/PD-L1 Blockade

### Applying Fecal Microbiota Transplantation in Anti-PD1/PD-L1 Therapy

Aiming to achieve an enhanced therapeutic efficacy and abrogate the treatment-associated adverse events of PD1/PD-L1 blockade, several pilot interventional strategies are currently underway to probe into the feasibility in manipulating gut microbes in cancer patients based on prior microbiota modulatory experience applied in other disorders. Among all, fecal microbiota transplantation (FMT) is now one of the most well-established pathways in modifying gut microbiota composition (71).

FMT is defined as transferring the gut microbiome from the donor entirely contained in stool suspension to the recipient, aiming to recover the microbial ecosystem in non-responsive patients (72). Nevertheless, FMT was not commonly applied until it was first reported to successfully treat *Clostridium difficile* infection (CDI) in 1982 (73). Nowadays, FMT has been approved into standard guidelines for recurrent CDI treatment, with its effectiveness approaching over 90% (74). Moreover, it has been pointed out that FMT could also boost therapeutic efficacy in other intestinal-dysbiotic-derived diseases such as IBD, which has aroused interest in the potential of applying FMT in cancer management, especially for overcoming ICI-resistant cancers as well as ameliorating ICI-associated toxicities (72, 75, 76). For instance, FMT was proved to re-establish the microbial diversity both preclinically and clinically, presumably *via* preventing hepatic necrosis progression and ameliorating cognitive function to improve hepatic encephalopathy status (77). Besides, FMT from healthy individuals could also significantly relieve radiotherapy-induced enteritis in irradiated mice by reorganizing the microbial ecosystem, thus improving the overall survival status (78). More importantly, concerning the previous supportive role of FMT in the ICI-nonresponsive cohort (79), FMT may represent a promising approach to facilitate ICI therapy. Baruch et al. investigated the safety and efficacy of introducing FMT in ten melanoma patients who suffered from PD1-refractory metastases when re-applying anti-PD1 therapy. Three out of ten displayed clinical responses, with two presented with a complete response and one partial response, potentially mediated through gene expression profile shifting and immune cell mobilization in both intestinal mucosa and TME (80). Meanwhile, Davar et al. claimed that patients carrying PD1-resistant melanoma could benefit from a combinatory approach of FMT and anti-PD1 therapy primarily through modulating microbiota composition and reprogramming the immune tone (81). Furthermore, FMT is also believed to abrogate immunotherapy-related toxicities. For example, supplementation of a cocktail of “beneficial bacteria” including *Bacteroidales* and *Burkholderiales via* FMT has been reported to relieve the ICI-induced colitis in antibiotic-exposed mice (82). Taken all, more ongoing clinical trials incorporating modulation of microbiota *via* FMT in cancer therapies are now in their infant stage, though several clinical tests have exhibited much excitement (NCT04130763, NCT04056026, NCT03341143, etc.) (83).

Despite all these hopes, the usage of FMT is still confined with several limitations. Firstly, transferring fecal content from donor to recipient may pose great risks for infection, even causing death (75). Although FMT is proposed to lessen ICI-induced toxicities with fewer side effects, the minimal sample size and insufficient mechanistic explanation appeal for further validation (84). Moreover, FMT is clouded by uncertainties concerning the undefined boundary of beneficial bacteria (24). When performing FMT, disease-promoting bacteria and antimicrobial pathogens could also be accidentally transmitted to the recipient, leading to unanticipated secondary effects such as obesity and carcinogenesis (24, 85). As such, extensive microbial screening regardless of bacteria, viruses, and parasites before conducting FMT would help to reduce the risks. Moreover, optimal donor choosing and proper sequencing, cultivating, and encapsulating skills for biologically activated commensals are also noteworthy taking into account. Also, a series of inclusion criteria should be considered, including but not limited to safety, standardized delivery methods, optimal FMT regimens, desired FMT duration, and basal host immunity variations (71). Hence, further microbial profiling of the donors and recipients as well as clearly illustrated mechanistic insights are crucial to achieving the maximal clinical value of FMT (85, 86).

### Administration of Probiotics as a Potential Tool in PD1/PD-L1 Blockade

There are also growing numbers of trials investigating the feasibility of oral administration of live bacteria consortia or putative beneficial commensals encapsulated inside the pills in the form of probiotics (87). In general, probiotics refer to groups of bacteria that benefit human health when ingested in sufficient amounts, with the advantage of simplicity, portability, and applicability. First hypothesized by Élie Metchnikoff in the early 1900s, probiotics were aimed to replace the harmful bacteria with beneficial ones, thus modulating gut microbial constitution and reversing immunosuppressive tone (88, 89). Basically, probiotics could exert beneficial effects *via* the following mechanisms: (1) confer colonization resistance against pathogenic bacterial strains *via* competing for nutrients and adhesion with pathogens, (2) stabilize the overall mucosal immunomodulation, (3) protect the integrity of the mucosal barrier *via* producing antimicrobial factors such as defensins and bacteriocins, (4) target and degrade gut toxins in the colon (90, 91).

Nevertheless, probiotics have been proposed to prevent and treat several diseases, their application in therapy still lacks consensus clarity. Early pre-clinical studies revealed the supportive role of administering probiotic mixture VSL#3 in mice undergoing chemotherapy, which showed substantial effectiveness in ameliorating irinotecan-induced diarrhea and weight loss (92). Furthermore, *A. muciniphila* administration was proved to boost the therapeutic effects in antibiotic-exposed mice receiving anti-PD1 therapy, and *Bifidobacterium* supplement largely reversed the anti-PD-L1 resistant status in melanoma-bearing mice, indicating its potential role in assisting PD1/PD-L1 blockade efficacy (20, 22). Recently, Tanoue et al. isolated 11 commensal strains that were capable of motivating IFN-γ producing T cells from the gut of germ-free mice, which were rarely found in human intestines. Feeding and recolonizing these strains significantly boosted anti-PD1 and anti-CTLA-4 therapeutic responses in tumor-bearing mice, indicating their synergistic role in assisting ICI therapy (93). Moreover, several pioneer trials applying probiotics for treating malignancies have been planned and implemented in action. For example, in trial NCT03817125, the orally delivered probiotics (SER-401) are supplied along with PD1 blockade therapy to metastatic melanoma patients, focusing on the clinical efficacy, potential toxicity, overall safety, host immune response alterations, as well as microbiota compositional change (71). A processing trial incorporates a diverse range of cancer patients, including melanoma, NSCLC, RCC, and bladder cancer candidates, employing defined bacteria strains (MRx0518) together with regular anti-PD1 ICI therapy, expecting to observe clinical efficacy (71). Another phase I trial aims to evaluate the effect of the probiotic *Clostridium butyricum* CBM588 strain in combination with nivolumab and ipilimumab in treating patients with kidney cancer (NCT03829111) (94).

Nonetheless, those probiotics that are deemed to be applied as dietary supplements are not strictly granted by the FDA review process before going into the market. In other words, less-regulated consumables lack definite scientific evidence, further indicating that their impact on gut flora and overall health may be pretty confined (95). Moreover, conflicting results also exist concerning its role in immunotherapy. In the study of Suez et al., providing an 11-strain probiotic cocktail resulted in delayed reconstitution of indigenous microbial flora instead of synergistic effect. In contrast, the application of FMT successfully reversed the dysbiotic state in the antibiotic-treated human cohort (96). What’s more, opportunistic probiotic translocation may appear in the circumstance of immunocompromised patients with a deficient and damaged intestinal barrier, which could pose risks for localized and systemic infectious complications, finally leading to bacteremia, endocarditis, pneumonia, and even death (89).

### The Potential Role of Diet and Prebiotics in Anti-PD1/PD-L1 Immunotherapy

As noted above, on the one hand, gut microbiota plays a key role in food digestion and nutrients absorption during dietary intake; on the other hand, profound changes in dietary consumption, in turn, leads to the microbiome compositional differentiation as well as their transcriptomics and metabolomic profile alterations to meet the energy needs (71, 87). Evidence has claimed that certain bacteria and associated metabolites displayed distinct responses in reply to particular nutrient intake and immunological stimulations, offering preliminary diet manipulation strategies to regulate commensal microbial diversity and strengthen host immunity (71, 87, 89). In particular, prebiotics was defined firstly by Gibson and Roberfroid in 1995 as non-viable food components (mainly represented as fibers) that benefit overall health by synergizing the expansion of beneficial bacteria (24). Additionally, other substances, such as poly-unsaturated fatty acids (PUFAs) and polyphenols, have also been proposed to possess prebiotic potential. Although prebiotics could promote the proliferation of specific beneficial bacteria consortiums, their effectiveness is largely dependent on the residual commensals inside the host; therefore, it is suggested to be used in the form of symbiotics (89). In general, prebiotics function in the intestines mainly in four ways, including stimulating the growth and enrichment of specific probiotics to combat pathogens and modulate immune response (97), directly interacting with pathogens to prevent the colonization and adhesion of pathogens (98), being selectively fermented by colonized probiotics to produce bioactive postbiotics (e.g., SCFA) (99), being absorbed into intestinal cells to alter the gene expression profiles and enhance IFN-γ and IL-10 production in CD4^+^ T cells (100).

Meanwhile, intense dietary changes could result in the outgrowth of therapeutic-favorable bacteria, further modifying outcomes of ICI therapies (87). According to David et al., it was demonstrated that a tendency towards high fat, low fiber-based diet would negatively impact the variety of the bacteria community, with a significant reduction in the number of *F. prausnitzii* that facilitate the metabolism of dietary plant polysaccharides found in the fecal samples of healthy individuals (101). In addition, RamirezFarias et al. claimed that the plant polysaccharide inulin was positively correlated with the selection of beneficial bacteria *Faecalibacterium* and *Bifidobacterium* species, the two taxa that were recently indicated to be responsible for enhanced responsiveness to PD1/PD-L1 blockade therapy (102). Apart from that, most research also described a cancer-preventing role of prebiotics primarily *via* assisting in chemotherapy and reducing associated toxicities. Taper et al. observed a potentiated drug efficacy and improved toxicity tolerance in liver tumor-bearing mice treated with six chemotherapeutics (5-FU, doxorubicin, vincristine, CTX, MTX, cytarabine) together with inulin or oligofructose instead of treating with chemotherapy alone (103).

Tentative clinical trials on dietary manipulation towards cancer patients are currently underway. A trial termed “BE GONE” trial (NCT02843425) has already begun to investigate the dietary alterations in cancer cohorts, with the intervention of adding another half cup of beans per day into their daily diet, indeed, measuring the shifts in gut commensals and inferring possible effect on their routine therapies (49). Notably, the divergence in host genetic background may plausibly explain the different outcomes concerning prebiotic administration (beneficial or detrimental), further supporting the necessity of designing specific prebiotic regimens based on the interindividual variability in response to prebiotic administration (89). Although these studies stay at their initial stage, both are expected to provide valuable results on how dietary and lifestyle intervention would impact the gut microbial composition, immunological interaction, disease progression as well as the overall outcome of malignancies (49).

### Directly Using Postbiotics as a Potential Regulator in PD1/PD-L1 Blockade

Not restricted to ingestion of viable bacteria in the form of probiotics, the soluble byproducts and metabolites generated from microorganisms also exert bioactive roles on the host’s overall health, known as postbiotics (89). The most representative example would be SCFA, which is routinely produced during carbohydrate fermentation. For certain probiotic strains, it is hypothesized that the culture supernatants themselves are capable of displaying bioactive effects in place of administrating live bacteria, postulating that postbiotics, in some circumstances, may overcome the limitation of microbes and serve as a safer and more cost-effective option compared to probiotic intervention in anti-PD1/PD-L1 therapy (104, 105).

Up to now, the putative mechanisms underlying the bioactive role of postbiotics are clearly illustrated. For one thing, postbiotics are expected to alleviate colonic inflammation and preserve the gut barrier integrity. For instance, *Lactobacillus rhamnosus GG* derived protein, named p40, is believed to block cytokine-driven apoptosis of epithelial cells to restore the gut barrier function, thus mitigating intestinal injury and initiating protective immune responses (89, 105, 106). For another, selective postbiotics, such as ferrichrome extracted from *Lactobacillus casei* ATCC334 supernatant, could also exhibit potential tumoricidal effects *via* activating apoptotic-associated signaling axis in colorectal cancer cells (89). Additionally, postbiotics have been shown to facilitate anticancer therapeutic response as well. *Lactobacillus plantarum* supernatant was reported to potentiate the cytotoxicity of 5-FU activity on colonic cancer cells *in vitro*, primarily through inducing apoptosis, shortening survival rate, and prohibiting the stemness features of cancer cells (107). Moreover, supernatants from *E. coli Nissle* 1917 and *L. fermentum* BR11 were also illustrated to decrease the occurrence of 5-FU-induced mucositis (108).

Furthermore, it is challenging to isolate and match corresponding molecules that specifically contribute to the therapeutic effects due to their complex diversity and substantial metabolites mixture among bacteria. Although the investigation in the postbiotics has been a rapidly developing but relatively uncharted field, more safety and application profiles need to be claimed in clinical settings as this area is becoming more and more understood and matured (89).

## Conclusions

Cancer is growing as one of the most significant burdens in modern society. Whereas conventional therapies display multiple defects, PD1/PD-L1 blockade immunotherapy seems like a promising choice with favorable clinical efficacy and relatively mild adverse effects. Excitingly, it is suggested that microbiota could serve as a critical determinant in PD1/PD-L1 therapy and alter the therapeutic response as well as treatment-associated toxicity, which ultimately alters the overall outcomes of patients. Fortunately, the flexibility and modifiability features of gut microbes provide chances for clinical application as a potential modality facilitating cancer management. However, this field is relatively young, and there still remains a great deal of ambiguities and doubts to be solved. As studies reported the positive correlation between the enrichment of favorable bacteria and optimistic anti-PD1/PD-L1 response, the underlying processes of immunomodulation of these species need to be further clarified. While the intestinal bacterial mapping in the responders evidently differs from the non-responders, whether the microbiota composition could be regarded as a biomarker in predicting the consequence and prognosis of the ICI therapy requires validation in the subsequent prospective studies. Despite the existing discrepancies among studies, standardization should be reached regarding the methodology of fecal sample collection, DNA extraction, sequencing method, as well as isolated individual confounding variables for further investigation. Also, larger cohorts should be incorporated to discover potential markers for response evaluation. In addition, the interplay between microbiota and other immuno-oncology modalities other than PD1/PD-L1 inhibitors such as cancer vaccine, T cell-targeted therapy, and oncolytic virus therapy needs more investigation, which may help to illustrate whether the presence of beneficial consortium is applicable and overlapping in a broad spectrum of immunotherapy. Besides, hiding intrinsic microbial signals from fungi and viruses should also be explored. Furthermore, possible transmission modalities, including FMT, probiotics, diet, prebiotics, and postbiotics, are discussed herein to investigate the putative manipulation methods of gut microbiota. The choice of the most optimal delivery method with prolonged duration remains to be confirmed with additional tests; at the same time, its safety, effectiveness, and flexibility should also be comprehensively evaluated as well, thus making the most of the gut microbiota in PD1/PD-L1 blockade.

## Future Perspectives

So far, preclinical evidence has encouraged applying microbial manipulation as a therapeutic strategy to potentiate immune responses, and this approach is currently being tested in ongoing trials with considerable progress. Of note, the application of personalized microbiome therapy may be a promising clinical orientation to favor anti-PD1/PD-L1 blockade (89). In particular, the main point is to figure out the prerequisite for effective intervention. Since not all subjects respond equally to gut microbiota modulation, it highly depends on the baseline characteristics, including overall immune status, genetic background, gut barrier integrity, and microbial diversity. Consequently, the scheme of microbiota manipulation could be designed to fit the personalized microbial spectrum of particular patients. All in all, the ultimate goal of utilizing the microbiome is to both assist in anti-PD1/PD-L1 therapy and to reduce the risks for related toxicities. Thus, it is expected that microbiota intervention may become one of the following milestones for personalized and precise therapies for cancer treatment.

## Author Contributions

TC and ZL provided the concepts of this review and designed its framework. YZ conducted the research, selected the literature findings and wrote the manuscript. All authors edited the manuscript. All authors contributed to the article and approved the submitted version.

## Funding

This work was supported by grants from the National Natural Science Foundation of China (no. 82060638) and ‘Double 10−Thousand Plan’ of Jiangxi Province (Innovation and Technology Professionals as the High−End Talent).

## Conflict of Interest

The authors declare that the research was conducted in the absence of any commercial or financial relationships that could be construed as a potential conflict of interest.

## Publisher’s Note

All claims expressed in this article are solely those of the authors and do not necessarily represent those of their affiliated organizations, or those of the publisher, the editors and the reviewers. Any product that may be evaluated in this article, or claim that may be made by its manufacturer, is not guaranteed or endorsed by the publisher.

## References

[1] WHO Health Statistics and Information Systems. In Projections of Mortality and Causes of Death, 2016 to 2060. WHO (2018).

[2] Mattiuzzi C Lippi G. Current Cancer Epidemiology. J Epidemiol Glob Health (2019) 9(4):217–22. doi: 10.2991/jegh.k.191008.001 PMC7310786 31854162

[3] Farkona S Diamandis EP Blasutig IM. Cancer Immunotherapy: The Beginning of the End of Cancer? BMC Med [Internet] (2016) 14(1):1–18. doi: 10.1186/s12916-016-0623-5 PMC4858828 27151159

[4] Sun JY Lu XJ. Cancer Immunotherapy: Current Applications and Challenges. Cancer Lett (2020) 480:1–3. doi: 10.1016/j.canlet.2020.03.024 32229188

[5] Padma VV. An Overview of Targeted Cancer Therapy. Biomed (2015) 5(4):1–6. doi: 10.7603/s40681-015-0019-4 PMC4662664 26613930

[6] Topalian SL Weiner GJ Pardoll DM. Cancer Immunotherapy Comes of Age. J Clin Oncol (2011) 29(36):4828–36. doi: 10.1200/JCO.2011.38.0899 PMC3255990 22042955

[7] Kennedy LB Salama AKS. A Review of Cancer Immunotherapy Toxicity. CA Cancer J Clin (2020) 70(2):86–104. doi: 10.3322/caac.21596 31944278

[8] Mellman I Coukos G Dranoff G. Cancer Immunotherapy Comes of Age. Nature (2011) 480(7378):480–9. doi: 10.1038/nature10673 PMC3967235 22193102

[9] Drake CG. Basic Overview of Current Immunotherapy Approaches in Urologic Malignancy. Urol Oncol Semin Orig Investig (2006) 24(5):413–8. doi: 10.1016/j.urolonc.2005.08.013 16962493

[10] Davis ID. An Overview of Cancer Immunotherapy. Immunol Cell Biol (2000) 78(3):179–95. doi: 10.1046/j.1440-1711.2000.00906.x 10849106

[11] Fridman WH Zitvogel L Sautès-Fridman C Kroemer G. The Immune Contexture in Cancer Prognosis and Treatment. Nat Rev Clin Oncol (2017) 14(12):717–34. doi: 10.1038/nrclinonc.2017.101 28741618

[12] Sharma P Allison JP. Immune Checkpoint Targeting in Cancer Therapy: Toward Combination Strategies With Curative Potential. Cell (2015) 161(2):205–14. doi: 10.1016/j.cell.2015.03.030 PMC5905674 25860605

[13] Hegde PS Chen DS. Top 10 Challenges in Cancer Immunotherapy. Immunity (2020) 52(1):17–35. doi: 10.1016/j.immuni.2019.12.011 31940268

[14] Zitvogel L Daillère R Roberti MP Routy B Kroemer G. Anticancer Effects of the Microbiome and Its Products. Nat Rev Microbiol [Internet] (2017) 15(8):465–78. doi: 10.1038/nrmicro.2017.44 28529325

[15] Goubet AG Daillère R Routy B Derosa L M. Roberti P Zitvogel L. The Impact of the Intestinal Microbiota in Therapeutic Responses Against Cancer. Comptes Rendus - Biol (2018) 341(5):284–9. doi: 10.1016/j.crvi.2018.03.004 29631891

[16] Elkrief A Derosa L Zitvogel L Kroemer G Routy B. The Intimate Relationship Between Gut Microbiota and Cancer Immunotherapy. Gut Microbes [Internet] (2019) 10(3):424–8. doi: 10.1080/19490976.2018.1527167 PMC6546322 30339501

[17] Pickard JM Zeng MY Caruso R Núñez G. Gut Microbiota: Role in Pathogen Colonization, Immune Responses, and Inflammatory Disease. Immunol Rev (2017) 279(1):70–89. doi: 10.1111/imr.12567 28856738 PMC5657496

[18] Yi M Jiao D Qin S Chu Q Li A Wu K. Manipulating Gut Microbiota Composition to Enhance the Therapeutic Effect of Cancer Immunotherapy. Integr Cancer Ther (2019) 18:1534735419876351. doi: 10.1177/1534735419876351 31517538 PMC7242797

[19] Levy M Kolodziejczyk AA Thaiss CA Elinav E. Dysbiosis and the Immune System. Nat Rev Immunol (2017) 17(4):219–32. doi: 10.1038/nri.2017.7 28260787

[20] Sivan A Corrales L Hubert N Williams JB Aquino-Michaels K Earley ZM. Commensal Bifidobacterium Promotes Antitumor Immunity and Facilitates Anti-PD-L1 Efficacy. Science (80-) (2015) 350(6264):1084–9. doi: 10.1126/science.aac4255 PMC4873287 26541606

[21] Gopalakrishnan V Spencer CN Nezi L Reuben A Andrews MC Karpinets TV. Gut Microbiome Modulates Response to Anti-PD-1 Immunotherapy in Melanoma Patients. Science (80-) (2018) 359(6371):97–103. doi: 10.1126/science.aan4236 PMC5827966 29097493

[22] Routy B Le Chatelier E Derosa L Duong CPM Alou MT Daillère R. Gut Microbiome Influences Efficacy of PD-1-Based Immunotherapy Against Epithelial Tumors. Science (80-) (2018) 359(6371):91–7.doi: 10.1126/science.aan3706 29097494

[23] Jin Y Dong H Xia L Yang Y Zhu Y Shen Y. The Diversity of Gut Microbiome is Associated With Favorable Responses to Anti–Programmed Death 1 Immunotherapy in Chinese Patients With NSCLC. J Thorac Oncol (2019) 14(8):1371–89. doi: 10.1016/j.jtho.2019.04.007 31026576

[24] Fessler J Matson V Gajewski TF. Exploring the Emerging Role of the Microbiome in Cancer Immunotherapy. J ImmunoTher Cancer (2019) 30(1):1–20. doi: 10.1186/s40425-019-0574-4 PMC6471869 30995949

[25] Pavet V Portal MM Moulin JC Herbrecht R Gronemeyer H. Towards Novel Paradigms for Cancer Therapy. Oncogene (2011) 30(1):1–20. doi: 10.1038/onc.2010.460 20935674

[26] Li W Deng X Chen T. Exploring the Modulatory Effects of Gut Microbiota in Anti-Cancer Therapy. Front Oncol (2021) 11(April):1–11. doi: 10.3389/fonc.2021.644454 PMC8076595 33928033

[27] Yi M Yu S Qin S Liu Q Xu H Zhao W. Gut Microbiome Modulates Efficacy of Immune Checkpoint Inhibitors. J Hematol Oncol (2018) 11(1):1–10. doi: 10.1186/s13045-018-0592-6 29580257 PMC5870075

[28] Good-Jacobson KL Szumilas CG Chen L Sharpe AH Tomayko MM Shlomchik MJ. PD-1 Regulates Germinal Center B Cell Survival and the Formation and Affinity of Long-Lived Plasma Cells. Nat Immunol (2010) 11(16):535–42. doi: 10.1038/ni.1877 PMC2874069 20453843

[29] Keir ME Butte MJ Freeman GJ Sharpe AH. PD-1 and Its Ligands in Tolerance and Immunity. Annu Rev Immunol (2008) 26:677–704. doi: 10.1146/annurev.immunol.26.021607.090331 18173375 PMC10637733

[30] Liang SC Latchman YE Buhlmann JE Tomczak MF Horwitz BH Freeman GJ. Regulation of PD-1, PD-L1, and PD-L2 Expression During Normal and Autoimmune Responses. Eur J Immunol (2003) 33(10):2706–16. doi: 10.1002/eji.200324228 14515254

[31] Ghiotto M Gauthier L Serriari N Pastor S Truneh A Nunès JA. PD-L1 and PD-L2 Differ in Their Molecular Mechanisms of Interaction With PD-1. Int Immunol (2010) 22(8):651–60. doi: 10.1093/intimm/dxq049 PMC3168865 20587542

[32] Hirano F Kaneko K Tamura H Dong H Wang S Ichikawa M. Blockade of B7-H1 and PD-1 by Monoclonal Antibodies Potentiates Cancer Therapeutic Immunity. Cancer Res (2005) 65(3):1089–96. doi: 10.1093/intimm/dxq049 15705911

[33] McLaughlin J Han G Schalper KA Carvajal-Hausdorf D Pelekanou V Rehman J. Quantitative Assessment of the Heterogeneity of PD-L1 Expression in Non-Small-Cell Lung Cancer. JAMA Oncol (2016) 2(1):46–54. doi: 10.1001/jamaoncol.2015.3638 26562159 PMC4941982

[34] Schalper KA Carvajal-Hausdorf D McLaughlin J Velcheti V Chen L Sanmamed M. Clinical Significance of PD-L1 Protein Expression on Tumor-Associated Macrophages in Lung Cancer. J Immunother Cancer (2015) 3 (Suppl 2):P415. doi: 10.1186/2051-1426-3-S2-P415

[35] Vassilakopoulou M Avgeris M Velcheti V Kotoula V Rampias T Chatzopoulos K. Evaluation of PD-L1 Expression and Associated Tumor-Infiltrating Lymphocytes in Laryngeal Squamous Cell Carcinoma. Clin Cancer Res (2016) 22(3):704–13. doi: 10.1158/1078-0432.CCR-15-1543 26408403

[36] Schalper KA Velcheti V Carvajal D Wimberly H Brown J Pusztai L. *In Situ* Tumor PD-L1 mRNA Expression is Associated With Increased Tils and Better Outcome in Breast Carcinomas. Clin Cancer Res (2014) 20(10):2773–82. doi: 10.1158/1078-0432.CCR-13-2702 24647569

[37] Kythreotou A Siddique A Mauri FA Bower M Pinato DJ. Pd-L1. J Clin Pathol (2018) 71(3):189–94. doi: 10.1136/jclinpath-2017-204853 29097600

[38] Butte MJ Keir ME Phamduy TB Sharpe AH Freeman GJ. Programmed Death-1 Ligand 1 Interacts Specifically With the B7-1 Costimulatory Molecule to Inhibit T Cell Responses. Immunity (2007) 27(1):111–22. doi: 10.1016/j.immuni.2007.05.016 PMC2707944 17629517

[39] Brahmer JR Drake CG Wollner I Powderly JD Picus J Sharfman WH. Phase I Study of Single-Agent Anti-Programmed Death-1 (MDX-1106) in Refractory Solid Tumors: Safety, Clinical Activity, Pharmacodynamics, and Immunologic Correlates. J Clin Oncol (2010) 28(19):3167–75. doi: 10.1200/JCO.2009.26.7609 PMC4834717 20516446

[40] Rittmeyer A Barlesi F Waterkamp D Park K Ciardiello F von Pawel J. Atezolizumab Versus Docetaxel in Patients With Previously Treated Non-Small-Cell Lung Cancer (OAK): A Phase 3, Open-Label, Multicentre Randomised Controlled Trial. Lancet (2017) 389(10066):255–65. doi: 10.1016/S0140-6736(16)32517-X PMC6886121 27979383

[41] Topalian SL Sznol M McDermott DF Kluger HM Carvajal RD Sharfman WH. Survival, Durable Tumor Remission, and Long-Term Safety in Patients With Advanced Melanoma Receiving Nivolumab. J Clin Oncol (2014) 32(10):1020–30. doi: 10.1200/JCO.2013.53.0105 PMC4811023 24590637

[42] Wolchok JD Kluger H Callahan MK Postow MA Rizvi NA Lesokhin AM. Nivolumab Plus Ipilimumab in Advanced Melanoma. N Engl J Med (2013) 369(2):122–33. doi: 10.1056/NEJMoa1302369 PMC5698004 23724867

[43] Larkin J Chiarion-Sileni V Gonzalez R Grob JJ Cowey CL Lao CD. Combined Nivolumab and Ipilimumab or Monotherapy in Untreated Melanoma. N Engl J Med (2015) 373(1):23–34. doi: 10.1056/NEJMoa1504030 26027431 PMC5698905

[44] Postow MA Sidlow R Hellmann MD. Immune-Related Adverse Events Associated With Immune Checkpoint Blockade. N Engl J Med (2018) 378(2):158–68. doi: 10.1056/NEJMra1703481 29320654

[45] Khoja L Day D Wei-Wu Chen T Siu LL Hansen AR. Tumour- and Class-Specific Patterns of Immune-Related Adverse Events of Immune Checkpoint Inhibitors: A Systematic Review. Ann Oncol (2017) 28(10):2377–85. doi: 10.1093/annonc/mdx286 28945858

[46] Robert C Schachter J Long GV Arance A Grob JJ Mortier L. Pembrolizumab Versus Ipilimumab in Advanced Melanoma. N Engl J Med (2015) 372(26):2521–32. doi: 10.1056/NEJMoa1503093 25891173

[47] Zhang JY Yan YY Li JJ Adhikari R Fu LW. PD-1/PD-L1 Based Combinational Cancer Therapy: Icing on the Cake. Front Pharmacol (2020) 11. doi: 10.3389/fphar.2020.00722 PMC7247431 32528284

[48] Bultman SJ. The Microbiome and Its Potential as a Cancer Preventive Intervention. Semin Oncol (2016) 43(1):97–106. doi: 10.1053/j.seminoncol.2015.09.001 26970128 PMC4789109

[49] Gopalakrishnan V Helmink BA Spencer CN Reuben A Wargo JA. The Influence of the Gut Microbiome on Cancer, Immunity, and Cancer Immunotherapy. Cancer Cell [Internet] (2018) 33(4):570–80. doi: 10.1016/j.ccell.2018.03.015 PMC6529202 29634945

[50] Li W Deng Y Chu Q Zhang P. Gut Microbiome and Cancer Immunotherapy. Cancer Lett (2019) 447(January):41–7. doi: 10.1016/j.canlet.2019.01.015 30684593

[51] Rezasoltani S Yadegar A Asadzadeh Aghdaei H Reza Zali M. Modulatory Effects of Gut Microbiome in Cancer Immunotherapy: A Novel Paradigm for Blockade of Immune Checkpoint Inhibitors. Cancer Med (2021) 10(3):1141–54. doi: 10.1002/cam4.3694 PMC7897953 33369247

[52] Honda K Littman DR. The Microbiota in Adaptive Immune Homeostasis and Disease. Nature (2016) 535(7610):75–84. doi: 10.1038/nature18848 27383982

[53] Zheng D Liwinski T Elinav E. Interaction Between Microbiota and Immunity in Health and Disease. Cell Res (2020) 30(6):492–506. doi: 10.1038/s41422-020-0332-7 32433595 PMC7264227

[54] Shui L Yang X Li J Yi C Sun Q Zhu H. Gut Microbiome as a Potential Factor for Modulating Resistance to Cancer Immunotherapy. Front Immunol (2020) 10. doi: 10.3389/fimmu.2019.02989 PMC6978681 32010123

[55] Hapfelmeier S Lawson MAE Slack E Kirundi JK Stoel M Heikenwalder M. Reversible Microbial Colonization of Germ-Free Mice Reveals the Dynamics of IgA Immune Responses. Science (80-) (2010) 328(5986):1705–9. doi: 10.1126/science.1188454 PMC3923373 20576892

[56] Britton GJ Contijoch EJ Mogno I Vennaro OH Llewellyn SR Ng R. Microbiotas From Humans With Inflammatory Bowel Disease Alter the Balance of Gut Th17 and Rorγt+ Regulatory T Cells and Exacerbate Colitis in Mice. Immunity (2019) 50(1):212–24.e4. doi: 10.1016/j.immuni.2018.12.015 PMC6512335 30650377

[57] Ismail AS Severson KM Vaishnava S Behrendt CL Yu X Benjamin JL. γδ Intraepithelial Lymphocytes are Essential Mediators of Host-Microbial Homeostasis at the Intestinal Mucosal Surface. Proc Natl Acad Sci U S A (2011) 108(21):8743–8. doi: 10.1073/pnas.1019574108 PMC3102410 21555560

[58] Matson V Fessler J Bao R Chongsuwat T Zha Y Alegre ML. The Commensal Microbiome is Associated With Anti-PD-1 Efficacy in Metastatic Melanoma Patients. Science (80-) (2018) 359(6731):104–8. doi: 10.1126/science.aao3290 PMC6707353 29302014

[59] Routy B Gopalakrishnan V Daillère R Zitvogel L Wargo JA Kroemer G. The Gut Microbiota Influences Anticancer Immunosurveillance and General Health. Nat Rev Clin Oncol (2018) 15(6):382–96. doi: 10.1038/s41571-018-0006-2 29636538

[60] Mullins IM Slingluff CL Lee JK Garbee CF Shu J Anderson SG. CXC Chemokine Receptor 3 Expression by Activated CD8+ T Cells is Associated With Survival in Melanoma Patients With Stage III Disease. Cancer Res (2004) 64(21):7697–701. doi: 10.1158/0008-5472.CAN-04-2059 15520172

[61] Morrison DJ Preston T. Formation of Short Chain Fatty Acids by the Gut Microbiota and Their Impact on Human Metabolism. Gut Microbes (2016) 7(3):189–200. doi: 10.1080/19490976.2015.1134082 26963409 PMC4939913

[62] Peters S Kerr KM Stahel R. PD-1 Blockade in Advanced NSCLC: A Focus on Pembrolizumab. Cancer Treat Rev (2018) 62:39–49. doi: 10.1016/j.ctrv.2017.10.002 29156447

[63] Daillère R Vétizou M Waldschmitt N Yamazaki T Isnard C Poirier-Colame V. Enterococcus Hirae and Barnesiella Intestinihominis Facilitate Cyclophosphamide-Induced Therapeutic Immunomodulatory Effects. Immunity (2016) 45(4):931–43. doi: 10.1016/j.immuni.2016.09.009 27717798

[64] Viaud S Saccheri F Mignot G Yamazaki T Daillère R Hannani D. The Intestinal Microbiota Modulates the Anticancer Immune Effects of Cyclophosphamide. Science (80-) (2013) 342(6161):971–6. doi: 10.1126/science.1240537 PMC4048947 24264990

[65] Blacher E Levy M Tatirovsky E Elinav E. Microbiome-Modulated Metabolites at the Interface of Host Immunity. J Immunol (2017) 198(2):572–80. doi: 10.4049/jimmunol.1601247 28069752

[66] Geller LT Barzily-Rokni M Danino T Jonas OH Shental N Nejman D. Potential Role of Intratumor Bacteria in Mediating Tumor Resistance to the Chemotherapeutic Drug Gemcitabine. Science (80-) (2017) 357(6356):1156–60. doi: 10.1126/science.aah5043 PMC5727343 28912244

[67] Cremonesi E Governa V Garzon JFG Mele V Amicarella F Muraro MG. Gut Microbiota Modulate T Cell Trafficking Into Human Colorectal Cancer. Gut (2018) 67(11):1984–4. doi: 10.1158/1538-7445.AM2018-1001 29437871

[68] Dubin K Callahan MK Ren B Khanin R Viale A Ling L. Intestinal Microbiome Analyses Identify Melanoma Patients at Risk for Checkpoint-Blockade-Induced Colitis. Nat Commun (2016) 7:10391. doi: 10.1038/ncomms10391 26837003 PMC4740747

[69] Chaput N Lepage P Coutzac C Soularue E Le Roux K Monot C. Baseline Gut Microbiota Predicts Clinical Response and Colitis in Metastatic Melanoma Patients Treated With Ipilimumab. Ann Oncol (2017) 28(6):1368–79. doi: 10.1093/annonc/mdx108 28368458

[70] Frankel AE Coughlin LA Kim J Froehlich TW Xie Y Frenkel EP. Metagenomic Shotgun Sequencing and Unbiased Metabolomic Profiling Identify Specific Human Gut Microbiota and Metabolites Associated With Immune Checkpoint Therapy Efficacy in Melanoma Patients. Neoplasia (United States) (2017) 19(10):848–55. doi: 10.1016/j.neo.2017.08.004 PMC5602478 28923537

[71] Khan MAW Ologun G Arora R McQuade JL Wargo JA. Gut Microbiome Modulates Response to Cancer Immunotherapy. Dig Dis Sci (2020) 65(3):885–96. doi: 10.1007/s10620-020-06111-x PMC7678709 32067144

[72] Chen D Wu J Jin D Wang B Cao H. Fecal Microbiota Transplantation in Cancer Management: Current Status and Perspectives. Int J Cancer (2019) 145(8):2021–31. doi: 10.1002/ijc.32003 PMC6767494 30458058

[73] Schwan A Sjolin S Trottestam U Aronsson B. Relapsing Clostridium Difficile Enterocolitis Cured by Rectal Infusion of Homologous Faeces. Lancet (1983) 2(8354):845. doi: 10.1016/S0140-6736(83)90753-5 6137662

[74] Surawicz CM Brandt LJ Binion DG Ananthakrishnan AN Curry SR Gilligan PH. Guidelines for Diagnosis, Treatment, and Prevention of Clostridium Difficile Infections. Am J Gastroenterol (2013) 108(4):478–98. doi: 10.1038/ajg.2013.4 23439232

[75] Baruch EN Gaglani T Wargo JA. Fecal Microbiota Transplantation as a Mean of Overcoming Immunotherapy-Resistant Cancers – Hype or Hope? Ther Adv Med Oncol (2021) 13:17588359211045853. doi: 10.1177/17588359211045853 34603515 PMC8481703

[76] Rohlke F Stollman N. Fecal Microbiota Transplantation in Relapsing Clostridium Difficile Infection. Ther Adv Gastroenterol (2012) 5(6):403–20. doi: 10.1177/1756283X12453637 PMC3491681 23152734

[77] Wang WW Zhang Y Huang XB You N Zheng L Li J. Fecal Microbiota Transplantation Prevents Hepatic Encephalopathy in Rats With Carbon Tetrachloride-Induced Acute Hepatic Dysfunction. World J Gastroenterol (2017) 23(38):6983–94. doi: 10.3748/wjg.v23.i38.6983 PMC5658316 29097871

[78] Cui M Xiao H Li Y Zhou L Zhao S Luo D. Faecal Microbiota Transplantation Protects Against Radiation-Induced Toxicity. EMBO Mol Med (2017) 9(4):448–61. doi: 10.15252/emmm.201606932 PMC5376756 28242755

[79] Kang YB Cai Y. Faecal Microbiota Transplantation Enhances Efficacy of Immune Checkpoint Inhibitors Therapy Against Cancer. World J Gastroenterol (2021) 27(32):5362–75. doi: 10.3748/wjg.v27.i32.5362 PMC8409158 34539138

[80] Baruch EN Youngster I Ben-Betzalel G Ortenberg R Lahat A Katz L. Fecal Microbiota Transplant Promotes Response in Immunotherapy-Refractory Melanoma Patients. Science (80-) (2021) 371(6529):602–9. doi: 10.1126/science.abb5920 33303685

[81] Davar D Dzutsev AK McCulloch JA Rodrigues RR Chauvin JM Morrison RM. Fecal Microbiota Transplant Overcomes Resistance to Anti-PD-1 Therapy in Melanoma Patients. Science (80-) (2021) 371(6529):595–602. doi: 10.1126/science.abf3363 PMC8097968 33542131

[82] Vétizou M Pitt JM Daillère R Lepage P Waldschmitt N Flament C. Anticancer Immunotherapy by CTLA-4 Blockade Relies on the Gut Microbiota. Science (80-) (2015) 350(6264):1079–84. doi: 10.1126/science.aad1329 PMC4721659 26541610

[83] Wu J Wang S Zheng B Qiu X Wang H Chen L. Modulation of Gut Microbiota to Enhance Effect of Checkpoint Inhibitor Immunotherapy. Front Immunol (2021) 12. doi: 10.3389/fimmu.2021.669150 PMC8276067 34267748

[84] Wang Y Wiesnoski DH Helmink BA Gopalakrishnan V Choi K DuPont HL. Fecal Microbiota Transplantation for Refractory Immune Checkpoint Inhibitor-Associated Colitis. Nat Med (2018) 24(12):1804–8. doi: 10.1038/s41591-018-0238-9 PMC6322556 30420754

[85] Cheng WY Wu CY Yu J. The Role of Gut Microbiota in Cancer Treatment: Friend or Foe? Gut (2020) 69(10):1867–76. doi: 10.1136/gutjnl-2020-321153 PMC7497589 32759302

[86] Giles EM D’Adamo GL Forster SC. The Future of Faecal Transplants. Nat Rev Microbiol (2019) 17(12):719. doi: 10.1038/s41579-019-0271-9 31534208

[87] Helmink BA Khan MAW Hermann A Gopalakrishnan V Wargo JA. The Microbiome, Cancer, and Cancer Therapy. Nat Med [Internet] (2019) 25(3):377–88. doi: 10.1038/s41591-019-0377-7 30842679

[88] Mackowiak PA. Recycling Metchnikoff: Probiotics, the Intestinal Microbiome and the Quest for Long Life. Front Public Heal (2013) 1. doi: 10.3389/fpubh.2013.00052 PMC3859987 24350221

[89] Fong W Li Q Yu J. Gut Microbiota Modulation: A Novel Strategy for Prevention and Treatment of Colorectal Cancer. Oncogene (2020) 39(26):4925–43. doi: 10.1038/s41388-020-1341-1 PMC7314664 32514151

[90] Gogineni V K Morrow LE. Probiotics: Mechanisms of Action and Clinical Applications. J Probiotics Heal (2013) 1:101. doi: 10.4172/2329-8901.1000101

[91] Panebianco C Andriulli A Pazienza V. Pharmacomicrobiomics: Exploiting the Drug-Microbiota Interactions in Anticancer Therapies. Microbiome (2018) 6(1):92. doi: 10.1186/s40168-018-0483-7 29789015 PMC5964925

[92] Bowen JM Stringer AM Gibson RJ Yeoh ASJ Hannam S Keefe DMK. VSL3 Probiotic Treatment Reduces Chemotherapy-Induced Diarrhea and Weight Loss. Cancer Biol Ther (2007) 6(9):1449–54. doi: 10.4161/cbt.6.9.4622 17881902

[93] Tanoue T Morita S Plichta DR Skelly AN Suda W Sugiura Y. A Defined Commensal Consortium Elicits CD8 T Cells and Anti-Cancer Immunity. Nature (2019) 565(7741):600–5. doi: 10.1038/s41586-019-0878-z 30675064

[94] Villéger R Lopès A Carrier G Veziant J Billard E Barnich N. Intestinal Microbiota: A Novel Target to Improve Anti-Tumor Treatment? Int J Mol Sci (2019) 20(18):1–25. doi: 10.3390/ijms20184584 PMC6770123 31533218

[95] Kristensen NB Bryrup T Allin KH Nielsen T Hansen TH Pedersen O. Alterations in Fecal Microbiota Composition by Probiotic Supplementation in Healthy Adults: A Systematic Review of Randomized Controlled Trials. Genome Med (2016) 8(1):52. doi: 10.1186/s13073-016-0300-5 27159972 PMC4862129

[96] Suez J Zmora N Zilberman-Schapira G Mor U Dori-Bachash M Bashiardes S. Post-Antibiotic Gut Mucosal Microbiome Reconstitution Is Impaired by Probiotics and Improved by Autologous FMT. Cell (2018) 174(6):1406–23. doi: 10.1016/j.cell.2018.08.047 30193113

[97] Lindsay JO Whelan K Stagg AJ Gobin P Al-Hassi HO Rayment N. Clinical, Microbiological, and Immunological Effects of Fructo-Oligosaccharide in Patients With Crohn’s Disease. Gut (2006) 55(3):348–55. doi: 10.1136/gut.2005.074971 PMC1856087 16162680

[98] Monteagudo-Mera A Rastall RA Gibson GR Charalampopoulos D Chatzifragkou A. Adhesion Mechanisms Mediated by Probiotics and Prebiotics and Their Potential Impact on Human Health. Appl Microbiol Biotechnol (2019) 103(16):6463–72. doi: 10.1007/s00253-019-09978-7 PMC6667406 31267231

[99] Wong JMW De Souza R Kendall CWC Emam A Jenkins DJA. Colonic Health: Fermentation and Short Chain Fatty Acids. : J Clin Gastroenterol (2006) 40(3):235–43. doi: 10.1097/00004836-200603000-00015 16633129

[100] Ito H Takemura N Sonoyama K Kawagishi H Topping DL Conlon MA. Degree of Polymerization of Inulin-Type Fructans Differentially Affects Number of Lactic Acid Bacteria, Intestinal Immune Functions, and Immunoglobulin a Secretion in the Rat Cecum. J Agric Food Chem (2011) 59(10):5771–8. doi: 10.1021/jf200859z 21506616

[101] David LA Maurice CF Carmody RN Gootenberg DB Button JE Wolfe BE. Diet Rapidly and Reproducibly Alters the Human Gut Microbiome. Nature (2014) 505(7484):559–63. doi: 10.1038/nature12820 PMC3957428 24336217

[102] Ramirez-Farias C Slezak K Fuller Z Duncan A Holtrop G Louis P. Effect of Inulin on the Human Gut Microbiota: Stimulation of Bifidobacterium Adolescentis and Faecalibacterium Prausnitzii. Br J Nutr (2009) 101(4):541–50. doi: 10.1017/S0007114508019880 18590586

[103] Taper HS Roberfroid MB. Nontoxic Potentiation of Cancer Chemotherapy by Dietary Oligofructose or Inulin. Nutr Cancer (2000) 38(1):1–5. doi: 10.1207/S15327914NC381_1 11341034

[104] Konstantinov SR Kuipers EJ Peppelenbosch MP. Functional Genomic Analyses of the Gut Microbiota for Crc Screening. Nat Rev Gastroenterol Hepatol (2013) 10(12):741–5. doi: 10.1038/nrgastro.2013.178 24042452

[105] Miller PL Carson TL. Mechanisms and Microbial Influences on CTLA-4 and PD-1-Based Immunotherapy in the Treatment of Cancer: A Narrative Review. Gut Pathog (2020) 12:43. doi: 10.1186/s13099-020-00381-6 32944086 PMC7488430

[106] Yan F Polk DB. Characterization of a Probiotic-Derived Soluble Protein Which Reveals a Mechanism of Preventive and Treatment Effects of Probiotics on Intestinal Inflammatory Diseases. Gut Microbes (2012) 3(1):25–8. doi: 10.4161/gmic.19245 PMC3337122 22356855

[107] An J Ha EM. Combination Therapy of Lactobacillus Plantarum Supernatant and 5-Fluouracil Increases Chemosensitivity in Colorectal Cancer Cells. J Microbiol Biotechnol (2016) 26(8):1490–503. doi: 10.4014/jmb.1605.05024 27221111

[108] Prisciandaro LD Geier MS Butler RN Cummins AG Howarth GS. Probiotic Factors Partially Improve Parameters of 5-Fluorouracil-Induced Intestinal Mucositis in Rats. Cancer Biol Ther (2011) 11(7):671–7. doi: 10.4161/cbt.11.7.14896 21307648

